# Hypoxia inducible factor-1α mediates the profibrotic effect of albumin in renal tubular cells

**DOI:** 10.1038/s41598-017-15972-8

**Published:** 2017-11-20

**Authors:** Junping Hu, Weili Wang, Fan Zhang, Pin-Lan Li, Krishna M. Boini, Fan Yi, Ningjun Li

**Affiliations:** 1 Department of Pharmacology & Toxicology, Virginia Commonwealth University School of Medicine, Richmond, VA 23298 USA; 2 Department of Pharmacological and Pharmaceutical Sciences, College of Pharmacy, University of Houston, Houston, TX 77204 USA; 3 Department of Pharmacology, Shandong University School of Medicine, Jinan, Shandong P.R. China

## Abstract

Proteinuria is closely associated with the progression of chronic kidney diseases (CKD) by producing renal tubulointerstitial fibrosis. Over-activation of hypoxia inducible factor (HIF)-1α has been implicated in the progression of CKD. The present study tested the hypothesis that HIF-1α mediates albumin-induced profibrotic effect in cultured renal proximal tubular cells. Incubation of the cells with albumin (40 μg/ml) for 72 hrs significantly increased the protein levels of HIF-1α, tissue inhibitor of metalloproteinase (TIMP)-1 and collagen-I, which were blocked by HIF-1α shRNA. Albumin also stimulated an epithelial-mesenchymal transition (EMT) as indicated by the decrease in epithelial marker E-cadherin, and the increase in mesenchymal markers α-smooth muscle actin and fibroblast-specific protein 1. HIF-1α shRNA blocked albumin-induced changes in these EMT markers as well. Furthermore, albumin reduced the level of hydroxylated HIF-1α, indicating an inhibition of the activity of prolyl-hydroxylases, enzymes promoting the degradation of HIF-1α. An anti-oxidant ascorbate reversed albumin-induced inhibition of prolyl-hydroxylase activity. Overexpression of prolyl-hydroxylase 2 (PHD2) transgene, a predominant isoform of PHDs in renal tubules, to reduce HIF-1α level significantly attenuated albumin-induced increases in TIMP-1 and collagen-I levels. These results suggest that albumin-induced oxidative stress inhibits PHD activity to accumulate HIF-1α, which mediates albumin-induced profibrotic effects in renal tubular cells.

## Introduction

Renal interstitial fibrosis is closely correlated with the progression of chronic kidney diseases (CKD) to end-stage renal disease^1,2^. Proteinuria is suggested to play a crucial role in the progression of CKD by producing renal tubulointerstitial fibrosis and is regarded as a major predictive factor of CKD progression in humans^3–6^. Albumin is the predominant component of proteinuria and its level in the urine is directly related to the progression of CKD. Exposure of proximal tubular epithelial cells to albumin has been shown to stimulate extracellular matrix (ECM) proteins and profibrotic cytokines^6–9^. It has also been demonstrated that renal tubular epithelial cells through epithelial to mesenchymal transition (EMT) is an important resource of fibrogenic myofibroblasts and plays an important role in renal tubulointerstitial fibrosis^10–12^. Meanwhile, evidence has been displayed that albumin induces EMT in renal tubular cells^13–15^. Therefore, albumin-induced EMT and profibrotic actions in renal tubular cells may play a critical role during the progression of chronic kidney damage.

Hypoxia inducible factor (HIF)-1α is a transcription factor and has recently been found to be involved in chronic renal injuries^16–18^. HIF-1α is up-regulated in different CKD^16–19^. HIF-1α stimulates the accumulation of collagen by activating several profibrogenic factors, such as the tissue inhibitor of metalloproteinase (TIMP), plasminogen activator inhibitor (PAI) and connective tissue growth factor (CTGF)^20–22^. It has been demonstrated that genetic deletion of HIF-1α in renal epithelial cells inhibits the tubulointerstitial fibrosis in unilateral ureteral obstruction model^22^ and that stable overexpression of HIF-1α in renal tubular cells promotes tubulointerstitial fibrosis in mice with 5/6 nephrectomy^23^. Therefore, although up-regulation of HIF-1α is protective in acute kidney injury^19,24^, the long-term HIF-1α activation is injurious in CKD^18,19,23,25–27^. Interestingly, albumin has been shown to induce the expression of HIF-1α in renal tubular cells^28,29^, which indicates that HIF-1α may also play a role in albumin-induced damage of renal cells.

HIF prolyl-hydroxylases, also named prolyl hydroxylase domain-containing proteins (PHDs), are the major enzymes to promote the degradation of HIF-1α^30^. PHDs catalyze site-specific proline hydroxylation of HIF-1α. Prolyl-hydroxylated HIF-1α is then captured by von Hippel-Lindau tumor suppressor protein (VHL), a component of an E3 ubiquitin ligase complex, and degraded by the ubiquitin-proteasome pathway. Three isoforms of PHD, including PHD1, PHD2, and PHD3, have been identified^30,31^. PHDs are expressed in kidneys and shown to regulate renal HIF-1α levels^32–36^. The predominant isoform of PHDs is PHD2 in kidneys, especially in the proximal tubules^32–34,37^. Given the important role of HIF-1α in the fibrogenic process in renal cells in response to different insults, PHD2 may also participate in the fibrogenic processes. Indeed, our recent works have demonstrated that PHD2/HIF-1α pathway participates in the fibrogenic process induced by angiotensin II and TGF-β^37,38^. The present study tested the hypothesis that PHD2/HIF-1α pathway mediates albumin-induced EMT and fibrogenic action in renal tubular cells.

## Materials and Methods

### Cell culture and experimental treatments

Experiments were performed in NRK-52E cells, a rat renal proximal tubular cell line from ATCC. For experiment, cells were transfected with different plasmids and treated with rat albumin (Sigma-Aldrich, Cat #A6414) for 72 hours (40 μg/ml), and then harvested for the isolation of RNA and protein. Some cells were incubated with H_2_O_2_ (5 × 10^−5^ M) and/or ascorbate (10^−4^ M) for 72 hours. The albumin concentration used in the current study was to mimic the concentration in tubular fluid based on the urinary albumin levels (at mg/ml range) in rat models of chronic kidney damage^39–42^ and the general knowledge that less than 1% of the glomerular filtrate (initial tubular fluid) is excreted as urine. The albumin concentration used was the concentration that produced significant changes in profibrotic factors in preliminary experiments. The albumin concentration in the current study was lower than that (at mg/ml range) in many other reports, which mimicked the albumin concentration in urine. We believe that the concentration used in the current study is more relevant.

### Transfection of plasmids into the cells

Plasmids expressing HIF-1α shRNA, HIF-1α transgene, PHD2 transgene or PHD2 shRNA were transfected into the cells using lipids DOTAP/DOPE (Avanti Polar Lipids, Alabaster, AL) as we described previously^38^. In brief, 5 µg of DNA was mixed with lipid solution in a ratio of 1:10 (DNA/lipid, w/w) in serum-free culture medium (5 ml for a 10 cm dish). Cells were incubated with this transfection medium for 6 h and then switched to normal medium for another 16 h. The cells were then ready for experiment. Luciferase plasmids were used in control cells.

### Western blot analysis

Extraction of cytosolic and nuclear protein and Western blot were performed as we described previously^37,38,43^. The cytosolic protein was used for Western blot analyses of tissue inhibitors of metalloproteinase (TIMP)-1, collagen I, PHD2 and hydroxylated HIF-1α (HIF-1α-OH). The nuclear fraction was used for Western blot analyses of HIF-1α. Briefly, after boiled for 10 min at 95 °C in a 5× loading buffer, cytosolic protein and nuclear protein were subjected to SDS-PAGE, transferred onto a PVDF membrane, blocked by solution with dry milk, and then the membrane was probed with primary antibodies of anti-rat collagen I (rabbit polyclonal, Calbiochem, 1:1000 dilution), anti-TIMP-1 (mouse monoclonal, R&D Systems, 1:1000), anti-PHD2 (rabbit polyclonal, Novus Biologicals, 1:500), anti-HIF-1α-OH (rabbit polyclonal, Novus Biologicals, 1:1000), and anti-HIF-1α (mouse monoclonal, Novus Biologicals, 1:300) overnight at 4 °C, respectively, followed by incubation with horseradish peroxidase-labeled secondary antibody (1:5000); β-actin was detected by using horseradish peroxidase-labeled anti-β-actin antibody (1:5000, Santa Cruz Biotechnology) as a loading control. Immunoreactive bands were detected by chemiluminescence methods and visualized on Kodak Omat X-ray films. The densitometry analyses of the blots were performed using Image J software (NIH) and the ratios of target proteins to β-actin were calculated. The values in control group were averaged and then all the ratios of band intensities were normalized to the mean value of control group.

### RNA extraction and quantitative RT-PCR analysis

Total RNA was extracted (TRIzol solution, Life Technologies, Inc. Rockville MD) and reverse-transcribed (RT) (cDNA Synthesis Kit, Bio-Rad, Hercules, CA), and then the RT products were amplified using a TaqMan Gene Expression Assays kit (Applied Biosystems), as we described previously^37,38^. A kit for detecting the levels of 18 S ribosomal RNA was used as an endogenous control. The relative gene expressions were calculated in accordance with the ΔΔCt method. Relative mRNA levels were expressed by the values of 2^−ΔΔCt^.

### Immunofluorescent microscopy

After fixation with 4% paraformaldehyde and permeabilization with 0.1% Triton X-100, cells cultured on glass coverslips were incubated with antibodies against fibroblast-specific protein (FSP)-1 (Abcam, 1:50), α-smooth muscle actin (α-SMA, Abcam, 1:100) or E-cadherin (E-Cad, Abcam, 1:100) at 4 °C overnight, respectively, then incubated with corresponding Alexa Fluor 555-coupled secondary antibodies at room temperature for 1 h, and at last, mounted and subjected to examinations using a confocal laser scanning microscope (Fluoview FV1000, Olympus, Japan). Integrated optical intensity (IOD) was calculated by using Image-pro plus v6.0 software (Media Cybernetics, Silver Spring, MD). The IOD values in control group were averaged, and all the IOD values normalized to the mean value of the control group.

### Statistics

Data are presented as means ± S.E.M. Significant differences between and within multiple groups were examined using ANOVA for repeated measures, followed by Tukey’s multiple-range test. *P* < 0.05 was considered statistically significant.

## Results

### Effects of HIF-1α shRNA on albumin-induced increases of collagen I, TIMP-1, proliferating cell nuclear antigen (PCNA), and Vimentin

**Figure 1:**
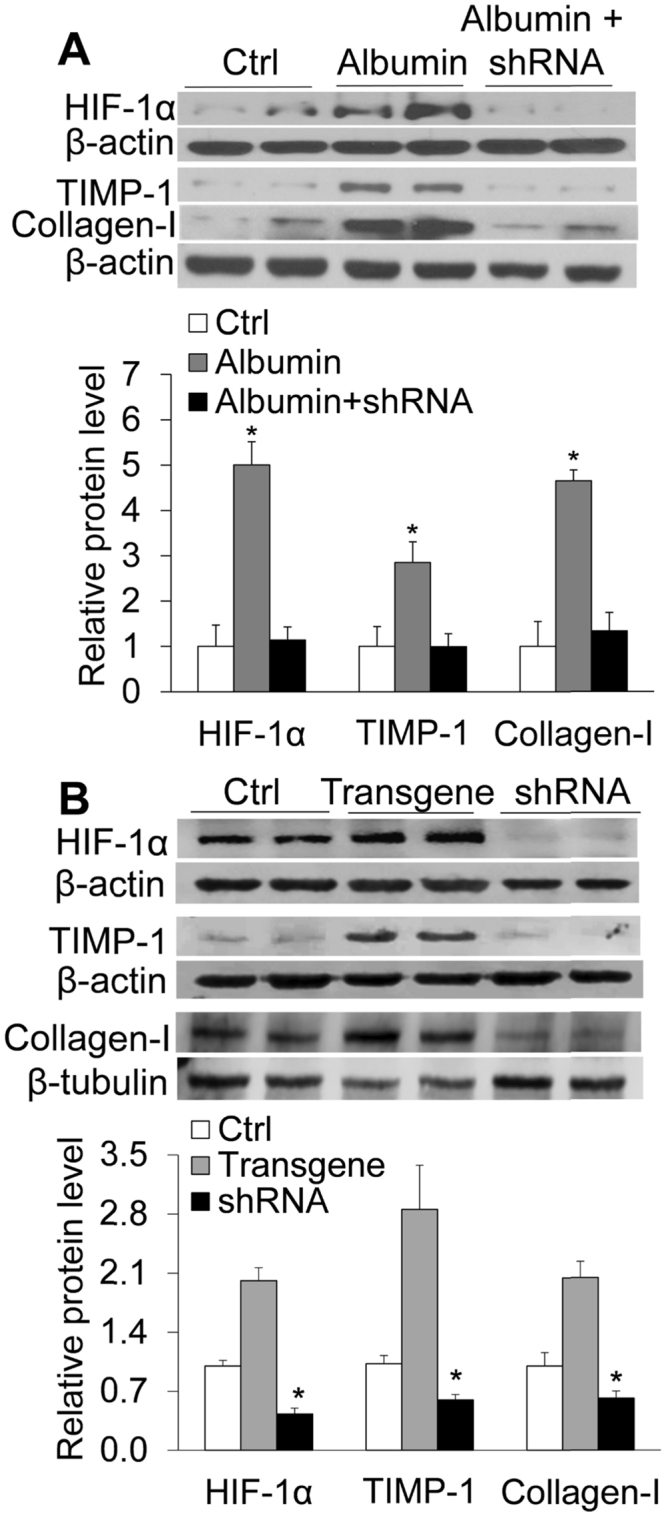
(**A**) Effect of HIF-1α shRNA on albumin-induced increase in the protein levels of HIF-1α, TIMP-1 and collagen-1 by Western blot analysis. Upper panel: Representative gel documents; Lower panel: Summarized data showing band intensity ratio to β-actin normalized to the value in Ctrl. Albumin, cells treated with albumin + control plasmids. Albumin + shRNA, cells treated with albumin + plasmids expressing HIF-1α shRNA. (**B**): Effect of HIF-1α transgene or shRNA on the protein levels of HIF-1α, TIMP-1 and collagen-1 by Western blot analysis. Upper panel: Representative gel documents; Lower panel: Summarized data. Ctrl, cells treated with control plasmids; HIF-1α, cells treated with plasmids expressing HIF-1α; shRNA, cells treated with plasmids expressing HIF-1α shRNA. n = 6 batches of cells, *P < 0.05 vs. other groups.

Treatment of albumin increased the protein level of HIF-1α and at the same time increased protein levels of collagen I and TIMP-1 in renal tubular cells (Fig. 1A). Transfection of HIF-1α shRNA into the cells blocked the increase of HIF-1α and at the same time abolished the albumin-induced increase of collagen I and TIMP-1 (Fig. 1A). These results suggest that albumin induces the accumulation of collagen I and TIMP-1 via the activation of HIF-1α. In cells without albumin treatment, HIF-1α transgene also significantly increased levels of collagen I and TIMP-1 and that HIF-1α shRNA reduced the levels of collagen I and TIMP-1 (Fig. 1B). These data demonstrated that using HIF-1α transgene to mimic the albumin-induced HIF-1α similarly upregulated the levels of collagen I and TIMP-1, further supporting that HIF-1α is the mediator in albumin-induced profibrotic effects.

**Figure 2:**
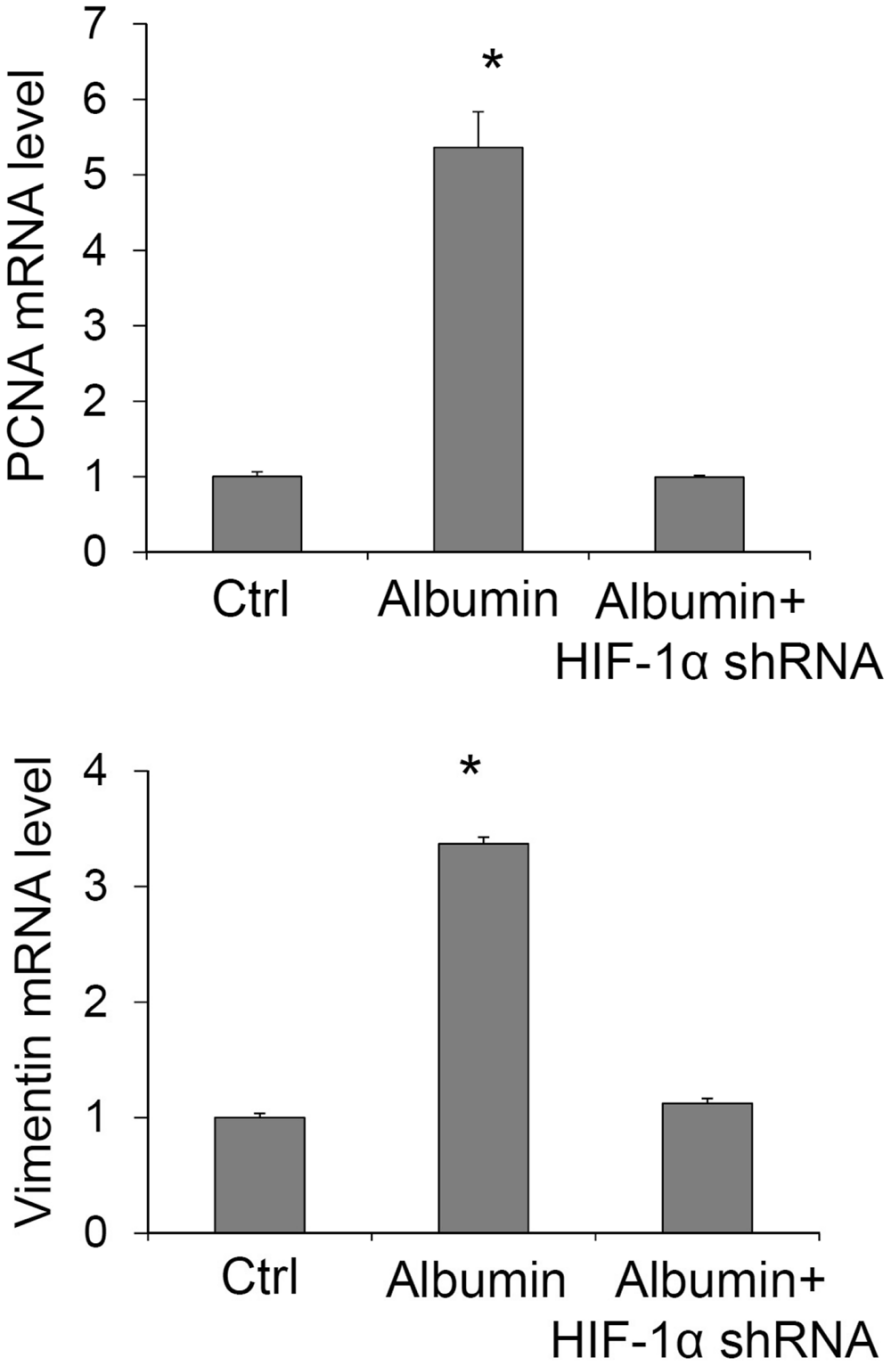
Effect of HIF-1α shRNA on albumin-induced increase in mRNA levels of PCNA and vimentin by Real-time RT-PCR analysis. Ctrl, cells treated with control plasmids. Albumin, cells treated with albumin + control plasmids. Albumin + HIF-1α shRNA, cells treated with albumin + plasmids expressing HIF-1α shRNA. n = 6 batches of cells, *P < 0.05 vs. other groups.

In addition, treatment of albumin significantly increased the mRNA levels of PCNA, a proliferation marker, and vimentin, an EMT marker. HIF-1α shRNA also blocked the increase of mRNA expression of PCNA and vimentin induced by albumin (Fig. 2). These results indicate that HIF-1α mediates the proliferation and transdifferentiation induced by albumin in renal tubular cells.

### Effects of HIF-1α shRNA on albumin-induced changes in the immunostaining of α-smooth muscle actin (α-SMA), fibroblast-specific protein (FSP)-1 and E-cadherin

**Figure 3:**
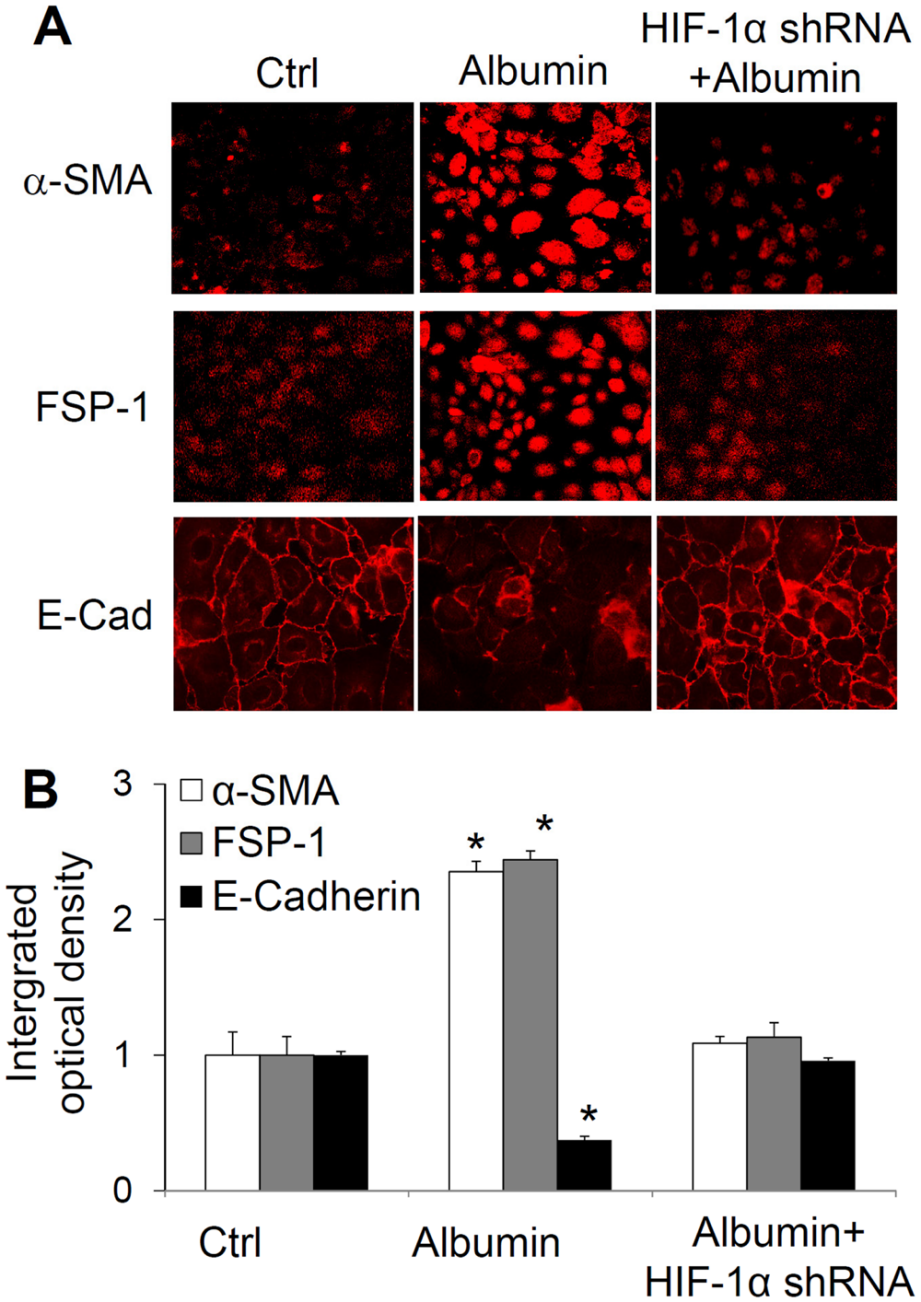
Effect of HIF-1α shRNA on albumin-induced changes in the immunostaining of α-SMA, FSP-1 and E-cadherin. Upper panel: Representative confocal images showing the immunostaining of α-SMA, FSP-1 and E-cadherin. Lower panel: Summarized integrated optical intensity of the fluorescent staining. Ctrl, cells treated with control plasmids. Albumin, cells treated with albumin + control plasmids. Albumin + HIF-1α shRNA, cells treated with albumin + plasmids expressing HIF-1α shRNA. n = 6 batches of cells, *P < 0.05 vs. other groups.

Figure 3 shows the immunostaining of α-SMA, FSP-1 and E-cadherin. The integrated optical intensity (IOD) of α-SMA and FSP-1, two mesenchymal markers, was weak in control cells and significantly increased in albumin-treated cells. In contrast, the staining intensity of E-cadherin, an epithelial marker, was strong in control cells and significantly weakened in albumin-treated cells. The staining pattern of E-cadherin was also disarranged in albumin-treated cells. These changes in mesenchymal and epithelial markers indicated an EMT process induced by albumin. However, in cells treated with HIF-1α shRNA the albumin-induced changes in these EMT markers were dramatically blocked (Fig. 3). These data suggest that albumin-induced EMT is mediated by the activation of HIF-1α in renal tubular cells.

### Effects of manipulating PHD2 gene on the levels of HIF-1α, collagen I and TIMP-1

**Figure 4:**
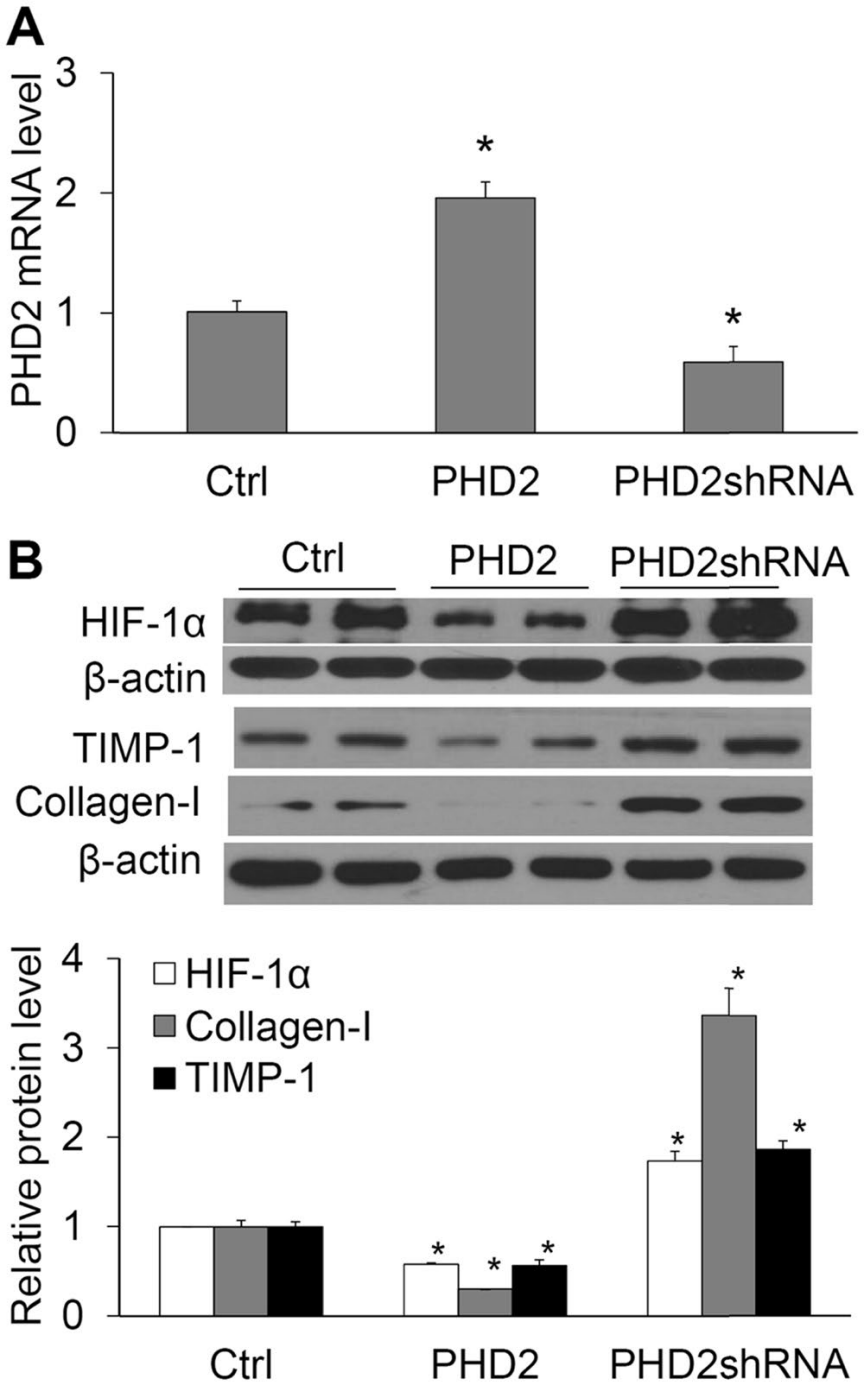
Effect of PHD2 transgene or PHD2 shRNA on the mRNA levels of PHD2 and the protein levels of HIF-1α, collagen I and TIMP-1. (**A**) The relative mRNA levels of PHD2 by Real-time RT-PCR analysis. (**B**) Protein levels of HIF-1α, collagen I and TIMP-1. Upper panel: Representative gel documents. Lower panel: Summarized data showing band intensity ratio of HIF-1α, collagen I and TIMP-1 to β-actin normalized to the value in Ctrl. Ctrl, cells treated with control plasmids. PHD2, cells treated with plasmids expressing PHD2. PHD2 shRNA, cells treated with plasmids expressing PHD2 shRNA. n = 6 batches of cells, *P < 0.05 vs. other two groups.

The data from PCR analysis showed that overexpression of PHD2 transgene significantly increased PHD2 mRNA level and silencing PHD2 gene by PHD2 shRNA decreased PHD2 mRNA level (Fig. 4A), which verified the successful manipulation of PHD2 gene expression. Overexpression of PHD2 transgene remarkably decreased the protein levels of HIF-1α, and at the same time, reduced levels of collagen I and TIMP-1 (Fig. 4B). In contrast, gene silencing by PHD2 shRNA increased the protein levels of HIF-1α, which was accompanied by the increased levels of collagen I and TIMP-1. These results suggest that PHD2 participates in the regulation of fibrogenic factors via regulating HIF-1α in renal tubular cells.

### Effects of manipulating PHD2 gene on albumin-induced increases in collagen I and TIMP-1

**Figure 5:**
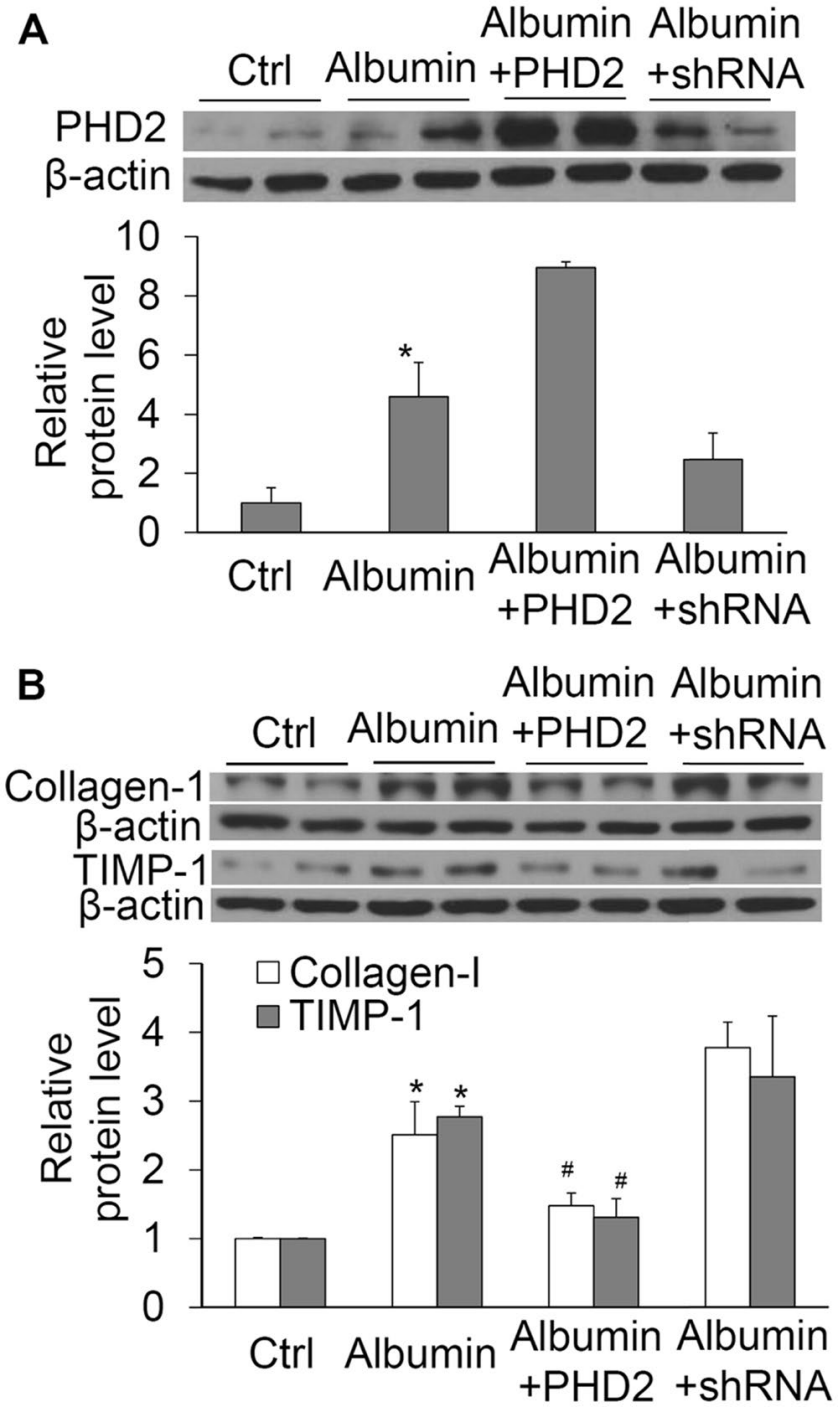
Effect of PHD2 transgene or PHD2 shRNA on albumin-induced increases in the levels of PHD2, collagen I and TIMP-1 by Western blot analysis. (**A**) PHD2, *P < 0.05 vs. all other groups. (**B**) collagen I and TIMP-1, *P < 0.05 vs. Ctrl and Albumin + PHD2. ^#^P < 0.05 vs. Albumin and Albumin + shRNA. n = 6 batches of cells.

Figure 5A shows results that verified the manipulation of PHD2 levels by PHD2 transgene or shRNA. Figure 5B shows that albumin-induced increases in the protein levels of collagen I and TIMP-1 were significantly reduced in cells transfected with plasmids expressing PHD2 transgene. In contrast, silencing of PHD2 gene showed a tendency of increase in the levels of collagen I and TIMP-1 induced by albumin, although there was no statistical significance. Taken together, these results indicate that PHD2 participates in the actions of albumin on collagen I and TIMP-1.

### Effect of albumin and antioxidant ascorbate on levels of prolyl hydroxylated HIF-1α, TIMP-1 and collagen-1

**Figure 6:**
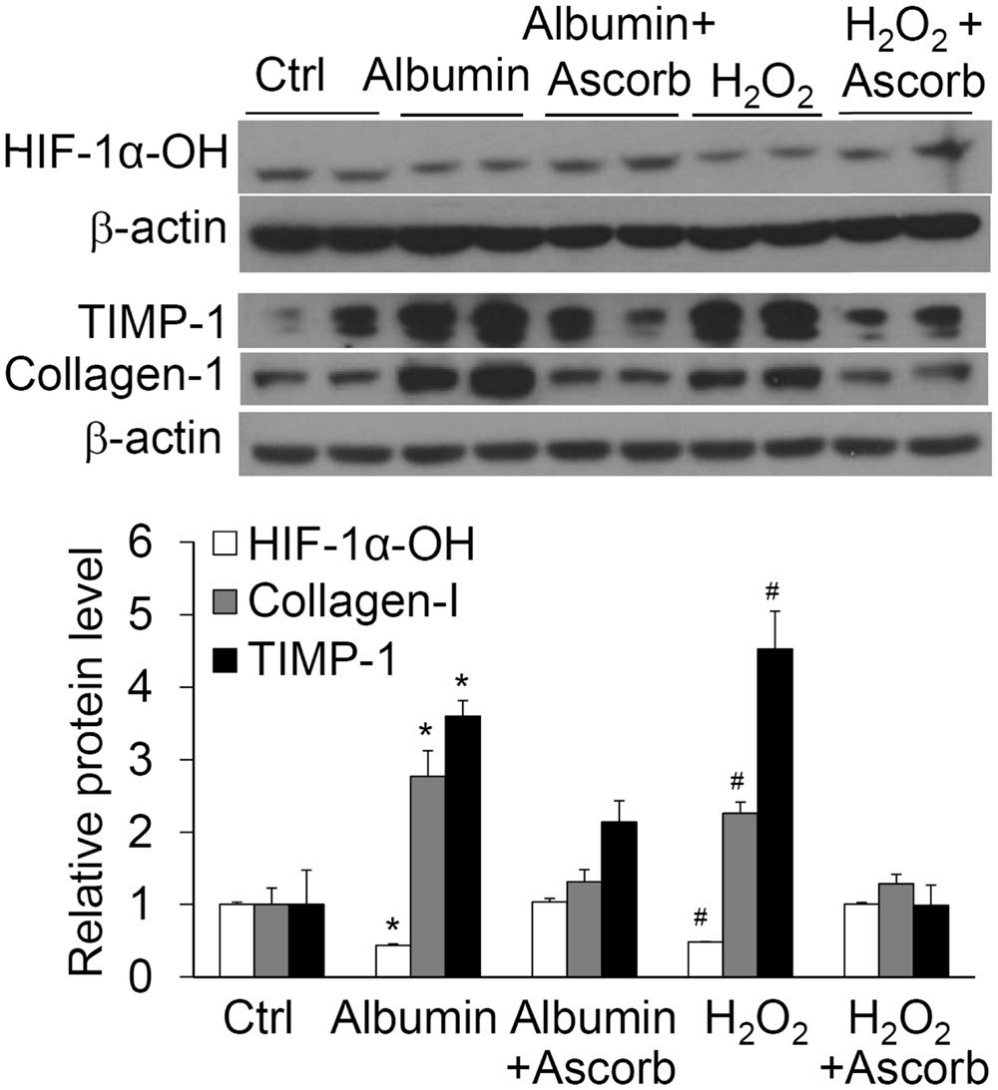
Effect of albumin or H_2_O_2_ on the levels of HIF-1α-OH, collagen I and TIMP-1 in the presence or absence of ascorbate by Western blot analysis. Upper panel: Representative gel documents; Lower panel: summarized data. *P < 0.05 vs. Ctrl and Albumin + Ascorbate; ^#^P < 0.05 vs. H_2_O_2_ + Ascorbate. n = 6 batches of cells.

The above data showed that albumin increased PHD2 protein level (Fig. 5A), indicating that albumin-induced increase in HIF-1α is not through reducing the levels of PHD2. We investigated whether albumin inhibited the activity of PHD, thereby increasing the levels of HIF-1α. The levels of prolyl hydroxylate HIF-1α (HIF-1α-OH) represent PHD activities. As shown in Fig. 6, albumin significantly decreased HIF-1α-OH levels, suggesting that albumin inhibits the function of PHD. When cells were incubated with ascorbate, an anti-oxidant, albumin-induced decrease in HIF-1α-OH protein level was blocked (Fig. 6), indicating that the inhibition of PHD activity is through albumin-induced oxidative stress. Incubation of the cells with H_2_O_2_ also reduced the levels of HIF-1α-OH, which was reversed by the treatment of ascorbate (Fig. 6). These data demonstrate that H_2_O_2_ mimics the effect of albumin on HIF-1α-OH, further suggesting that albumin-induced oxidative stress inhibited PHD activity. The changes of HIF-1α-OH levels were accompanied by the reverse changes of collagen I and TIMP-1 levels (Fig. 6). These data suggest that albumin increases TIMP-1 and induces collagen I through inhibition of PHD activity via oxidative stress in renal tubular cells.

## Discussion

The major findings of the present study include (1) silencing of HIF-1α gene to inhibit albumin-induced HIF-1α accumulation blocked albumin-induced increases in TIMP-1 and collagen I, suggesting that HIF-1α accumulation mediates albumin-induced fibrogenic effects in renal tubular cells; (2) silencing of HIF-1α gene inhibited albumin-induced increases in cell proliferation marker PCNA and mesenchymal markers vimentin, α-SMA and FSP-1, and also blocked albumin-induced decrease in epithelial marker E-cadherin, suggesting that accumulation of HIF-1α mediates the albumin-induced EMT; (3) overexpression of PHD2 transgene to reduce the level of HIF-1α inhibited albumin-induced increase in TIMP-1 and collagen-1, suggesting that PHD2 participates in albumin-induced fibrogenic effect; (4) albumin reduced the levels of HIF-1α-OH and that antioxidant ascorbate reversed the effects of albumin on HIF-1α-OH, collagen I and TIMP-1, suggesting that albumin increases the levels of HIF-1α and the fibrogenic factors through inhibition of PHD activity via oxidative stress in renal tubular cells.

The accumulation of ECM is the hallmark of tubulointerstitial fibrosis and that damage in renal proximal tubular cells is mostly responsible for the pathological accumulation of ECM in chronic kidney diseases^7,10–12,44^. Thus, proximal tubular cell line was used in the present study. Collagen is the main component of ECM deposition and that type I collagen is the most abundant member of the fibrillary collagen family^45–47^. Meanwhile, matrix metalloproteinases (MMPs) is a key family of proteases to degrade the components of ECM and that TIMP-1 is the preferential and most extensively studied tissue inhibitor of MMP involved in renal interstitial fibrosis^47–49^. Therefore, the present study used the increases of collagen I and TIMP-1 as indicators of albumin-induced profibrotic action. Although urinary protein, particularly albumin, is being recognized as a key mediator of renal tubulointerstitial injury in CKD^50^ and that proteinuria is one of the most common inducer of tubular EMT^51,52^, the mechanisms by which urinary protein produces tubulointerstitial fibrosis remain not clear. The results from the present study showed that albumin induced the accumulation of HIF-1α and increased the levels of collagen I and TIMP-1 and that silencing of HIF-1α gene blocked albumin-induced increases of collagen I and TIMP-1 levels in renal tubular cells. To our knowledge, these findings provide the first evidence that albumin-induced fibrogenic effect is mediated by HIF-1α accumulation and reveal a novel mechanism by which proteinuria produces fibrogenic actions.

Epithelial-to-mesenchymal transition (EMT) is a process by which epithelial cells lose their epithelial specific markers, undergo cytoskeletal remodeling, and gain a mesenchymal phenotype. More and more studies show that tubular EMT is an important resource of fibrogenic myofibroblasts and plays a central role in tubulointerstitial fibrosis^44,53^, such as in diabetes nephropathy^54^. During EMT, epithelial cells transdifferentiate into mesenchymal cells, levels of cytoskeletal proteins (e.g. α-SMA)^55,56^ and signal transduction proteins (e.g. FSP-1) are increased^55^, and that the expression of epithelial genes, including E-cadherin, is repressed^57^. Morphologically, the epithelial marker cadherin protein is delocalized from cell membrane during EMT^58^. Thus, the changes in α-SMA, FSP-1 and E-cadherin have been widely used as indicators for EMT^59^. Vimentins are class-III intermediate filaments found in various non-epithelial cells, especially mesenchymal cells. It is also often used as a marker of mesenchymal-derived cells or cell transdifferentiation. The expression and synthesis of proliferating cell nuclear antigen (PCNA) is linked with cell proliferation^60,61^. Thus, we also detected the changes of PCNA, vimentin, α-SMA, FSP-1 and E-cadherin to evaluate the role of HIF-1α in albumin-induced EMT. Our results displayed that HIF-1α shRNA blocked the changes in these EMT markers induced by albumin, suggesting that HIF-1α is involved in the relatively early stage of the fibrogenic process induced by albumin.

PHDs have been shown to degrade HIF-1α and thereby regulate the expression of HIF-1α target genes^62,63^. Given the findings in the present study that HIF-1α mediates the fibrogenic effect of albumin in renal tubular cells, we tested the hypothesis that PHDs may also participate in the fibrogenic actions of albumin in renal tubular cells. Indeed, the present study demonstrated that manipulating PHD2 changed the levels of TIMP-1 and collagen-1 and also altered the effects of albumin on TIMP-1 and collagen-1, suggesting that PHD2, via regulating HIF-1α, is an upstream signal in fibrogenesis and involved in albumin-induced fibrogenic effect in renal tubular cells. These results reveal that PHD2/HIF-1α may be a novel signaling pathway in chronic renal injury associated with proteinuria. Because our recent works have also demonstrated that PHD2/HIF-1α pathway participates in the fibrogenic processes induced by angiotensin II and TGF-β^37,38^, PHD2/HIF-1α pathway is probably the common molecular mechanism in the fibrogenic processes in response to different insults in renal cells.

Interestingly, our results showed that albumin increased both HIF-1α and PHD2 levels, which indicates that albumin-induced accumulation of HIF-1α is not a consequence of decreased PHD2 expression. Several inducers have been reported to activate HIF-1α by inhibiting PHD enzyme activity such as angiotensin II and thrombin^64^. We thus tested whether albumin stimulated HIF-1α via inhibiting the enzyme activity of PHD. Our results showed that albumin reduced the level of hydroxylated HIF-1α, demonstrating an inhibition of PHD activity by albumin. These data suggest that albumin increases HIF-1α level to promote fibrogenesis via inhibition of PHD activity.

However, how albumin reduced PHD enzyme activity remained to be answered. The activity of PHD is inhibited by low oxygen tension^31^ and also regulated by mechanisms independent of oxygen levels^65,66^, such as redox signals^38,65,66^. PHDs belong to the family of the Fe (II) and 2-oxoglutarate-dependent dioxygenase^67^. PHDs catalyze hydroxylation of HIF-1α using oxygen and 2-oxoglutarate as co-substrates as well as ascorbate and Fe^2+^ as cofactors. Oxidation promotes the conversion of Fe^2+^ to Fe^3+^ in the cells^64,68^. Reactive oxygen species (ROS), especially H_2_O_2_, have been evidenced to regulate PHD activity^64,68^. As albumin has been shown to stimulate ROS production and increase H_2_O_2_ in renal tubular cells^69,70^, we tested whether albumin inhibited PHD activity through oxidative stress and found that anti-oxidant reversed the inhibitory effect of albumin on PHD activity, suggesting that albumin inhibits PHD activity by inducing oxidant stress.

It should be pointed out that there are debates about the involvement of EMT in renal fibrosis *in vivo* ^71–75^. The supporting evidence includes the loss of epithelial markers, the acquisition of mesenchymal markers and the collagen synthesis by epithelial cells in diseased kidneys from both human patients and animal models, as well as the effective strategies based on EMT mechanism for the treatment of experimental fibrosis. The evidence against EMT *in vivo* includes failure to detect tubular epithelial cell–derived fibroblasts using lineage-tracing techniques and no EMT marker detected in labeled tubular cells in diseased kidneys. The arguments are that the detection of intermediate stages of EMT in injured kidney is the current gold standard and that to observe EMT process and cell migration in real time *in vivo* is not feasible with current technology. Nevertheless, our current study revealed the important role of HIF-1α in albumin-induced EMT in renal tubular cells.

In summary, the present study demonstrated that incubation of renal tubular cells with albumin increased the levels of HIF-1α and fibrogenic factors TIMP-1 and collagen-1, HIF-1α shRNA blocked the increases in TIMP-1 and collagen-1 and eliminated the changes in EMT markers induced by albumin, manipulating PHD2 levels altered the effect of albumin on TIMP-1 and collagen-1, albumin reduced the levels of hydroxylated HIF-1α and that an anti-oxidant reversed the inhibitory effect of albumin on hydroxylated HIF-1α. It is concluded that albumin activates HIF-1α and consequently produces fibrogenic effect through inhibition of PHD activity via oxidative stress in renal proximal tubule cells. Such redox regulation of PHD/HIF-1α pathway may constitute a novel pathogenic pathway mediating chronic renal injury under various pathological conditions associated with proteinuria.
